# Impact of Obesity-Associated SARS-CoV-2 Mutations on COVID-19 Severity and Clinical Outcomes

**DOI:** 10.3390/v17010038

**Published:** 2024-12-30

**Authors:** Ana B. Martínez-Martinez, Alexander Tristancho-Baró, Beatriz Garcia-Rodriguez, Marina Clavel-Millan, Maria Pilar Palacian, Ana Milagro, Antonio Rezusta, Jose M. Arbones-Mainar

**Affiliations:** 1 Facultad de Ciencias de la Salud, Universidad de Zaragoza, 50009 Zaragoza, Spain; amarmar@unizar.es; 2 Instituto de Investigación Sanitaria Aragón, 50009 Zaragoza, Spain; bea_garcia_rodriguez@hotmail.com (B.G.-R.); mclavelm@gmail.com (M.C.-M.); arezusta@salud.aragon.es (A.R.); 3 Department of Clinical Microbiology, Miguel Servet University Hospital, 50009 Zaragoza, Spain; alexander.tristancho1@gmail.com (A.T.-B.); ppalacian@salud.aragon.es (M.P.P.); anamilagro@gmail.com (A.M.); 4 Department of Clinical Biochemistry, Miguel Servet University Hospital, 50009 Zaragoza, Spain; 5 Adipocyte and Fat Biology Laboratory (AdipoFat), Instituto Aragonés de Ciencias de la Salud (IACS), 50009 Zaragoza, Spain; 6 CIBER Fisiopatología Obesidad y Nutrición (CIBERObn), Instituto Salud Carlos III, 28029 Madrid, Spain

**Keywords:** COVID-19, SARS-CoV-2, obesity, genetic mutations, virulence, vaccine development, immune response, hospitalization risk

## Abstract

This study explores the relationship between specific SARS-CoV-2 mutations and obesity, focusing on how these mutations may influence COVID-19 severity and outcomes in high-BMI individuals. We analyzed 205 viral mutations from a cohort of 675 patients, examining the association of mutations with BMI, hospitalization, and mortality rates. Logistic regression models and statistical analyses were applied to assess the impact of significant mutations on clinical outcomes, including inflammatory markers and antibody levels. Our findings revealed three key mutations—C14599T, A20268G, and C313T—that were associated with elevated BMI. Notably, C14599T appeared to be protective against hospitalization, suggesting context-dependent effects, while A20268G was linked to a 50% increase in hospitalization risk and elevated antibody levels, potentially indicating an adaptive immune response. C313T showed a 428% increase in mortality risk, marking it as a possible poor-prognosis marker. Interestingly, all three mutations were synonymous, suggesting adaptive roles in obesity-driven environments despite not altering viral protein structures. These results emphasize the importance of studying mutations within the broader context of comorbidities, other mutations, and regional factors to enhance our understanding of SARS-CoV-2 adaptation in high-risk groups. Further validation in larger cohorts is necessary to confirm these associations and to assess their clinical significance.

## 1. Introduction

The COVID-19 pandemic has posed significant and unprecedented challenges to global health systems [1]. Despite widespread public health measures and the development of vaccines, certain groups bear a disproportionate burden of severe COVID-19 outcomes [2,3]. Among these vulnerable groups, individuals with obesity have emerged as being particularly at risk [4]. Clinical studies consistently show that obesity is associated with an elevated risk of adverse COVID-19 outcomes, including higher rates of hospitalization, intensive care unit (ICU) admission, and mortality [5,6,7]. With over 650 million adults affected worldwide and obesity rates continuing to rise, understanding how SARS-CoV-2 interacts with this high-risk population is crucial to improving clinical outcomes and guiding public health interventions [8]. In addition to acute illness, long COVID has emerged as a pressing concern, with prolonged symptoms persisting well beyond the initial infection phase [9]. Recent studies indicate that individuals with obesity may be more susceptible to long COVID, leading to extended recovery times and placing additional burdens on healthcare systems [10,11,12,13]. Additionally, vaccination coverage remains inconsistent in many countries, with significant rates of undervaccination observed in some high-risk populations [14]. This is especially concerning for individuals with obesity, who are already predisposed to severe COVID-19 outcomes.

The mechanisms behind the association between obesity and severe COVID-19 outcomes are complex and likely multifactorial, involving immunological, metabolic, and physiological alterations linked to obesity. Obesity-induced chronic inflammation contributes to a proinflammatory environment characterized by elevated levels of cytokines such as interleukin (IL)-6, IL-1β, and tumor necrosis factor-alpha (TNF-α), which are implicated in the pathogenesis of COVID-19. This persistent inflammation may impair the immune system’s ability to mount an effective response, increasing susceptibility to severe infections and promoting conditions such as cytokine storm, a phenomenon strongly associated with adverse outcomes in COVID-19 patients [15,16].

Furthermore, adipose tissue in obese individuals serves as a reservoir for inflammatory cells, including macrophages and other innate immune cells, which secrete cytokines that exacerbate systemic inflammation. This low-grade chronic inflammation can disrupt the balance between innate and adaptive immune responses, impairing the function of T cells and other essential components of antiviral immunity [17]. Additionally, excess adipose tissue may increase the expression of angiotensin-converting enzyme 2 (ACE2), the receptor through which SARS-CoV-2 enters cells, potentially facilitating viral entry and replication, thereby increasing the viral load and worsening the disease severity [18].

Physiologically, obesity is associated with compromised lung function due to factors like reduced expiratory reserve volume and respiratory system compliance. These issues are exacerbated in the supine position, contributing to respiratory difficulties that may lead to acute respiratory distress syndrome (ARDS), a severe complication of COVID-19 [19,20].

Collectively, these factors not only worsen disease severity but also create an environment conducive to viral persistence, mutation, and adaptation, raising concerns about the potential for obesity to influence SARS-CoV-2 evolution [21]. The prolonged viral presence within an inflammatory environment theoretically provides SARS-CoV-2 with opportunities for genetic variation and mutation selection. However, the nature of the interaction between SARS-CoV-2 and the host environment in obesity remains unclear. Some studies suggest that the correlation between obesity and viral mutation frequency may be indirect, being influenced by comorbidities such as hypertension, diabetes, and cardiovascular disease [22,23,24].

Given these uncertainties, this study aims to characterize specific SARS-CoV-2 mutations associated with a higher BMI and to assess their influence on clinical outcomes in patients with obesity, seeking to clarify whether particular viral mutations contribute to the heightened risk observed in this growing population.

## 2. Materials and Methods

### 2.1. Study Design and Patient Selection

This study is an exploratory cross-sectional descriptive analysis involving 675 hospitalized COVID-19 patients at Hospital Universitario Miguel Servet (Spain). A total of 1159 patients were initially screened, and those with complete clinical and genetic information were included. Demographic and clinical data, such as age, gender, BMI, and hospitalization outcomes, were collected from medical records. To minimize selection bias, patients were recruited consecutively between March 2020 and February 2021, and standardized data collection protocols were employed. Potential confounders, such as age, gender, and comorbidities, were accounted for during the statistical analyses. Ethical approval was obtained from our regional ethics committee (CEIC-A, ref PI20/542).

### 2.2. Viral Genome Sequencing and Variant Calling

Nasopharyngeal swabs were collected from March 2020 to February 2021 and stored in guanidinium thiocyanate-free viral transport media. Samples positive for SARS-CoV-2 by PCR (cycle threshold values < 30 for all targets) underwent whole-genome next-generation sequencing (NGS). Viral RNA was extracted using a magnetic capture-based method on the Microlab STARlet^®^ system (Hamilton Company, Reno, NV, USA) following the manufacturer’s instructions, yielding 60 µL of eluate.

PCR detection utilized the Allplex™ 2019-nCoV Assay (Seegene Inc., Seoul, Republic of Korea), targeting the RdRP, N, and E genes. For sequencing, reverse transcription and amplification were performed using the ARTIC V3 protocol. Briefly, cDNA synthesis involved hexamers, dNTPs, RNaseOUT™ Recombinant Ribonuclease Inhibitor, and SuperScript IV™ Reverse Transcriptase (Invitrogen, Carlsbad, CA, USA), with incubation at 42 °C for 50 min and denaturation at 70 °C for 10 min. Amplification used multiplex PCR in two pools with the V3 primer set.

Libraries for sequencing were prepared using the Illumina^®^ Nextera DNA Flex™ kit (Illumina Inc., San Diego, CA, USA). The cDNA was fragmented, cleaned, indexed, and quantified using Qubit™ fluorometry (Thermo Fisher Scientific, Waltham, MA, USA) and Bioanalyzer™ (Agilent Technologies, Santa Clara, CA, USA). Sequencing was performed on Illumina^®^ iSeq™ and MiSeq™ platforms with paired-end protocols (300 cycles; 150 base pairs).

Samples were processed across three facilities: Instituto de Biomedicina de Valencia (75%), Hospital Universitario Miguel Servet (15%), and Centro Nacional de Microbiología Instituto de Salud Carlos III (10%), using standardized protocols.

FASTQ files were quality-assessed using fastqc v0.11.9 and trimmomatic v0.22, removing reads with quality scores below Q28. Genome, consensus generation, and lineage assignment were performed using the HaVoC pipeline v1 with NC_045512.2 as the reference genome. Variant calling and annotation were conducted with Nextclade v1.2.2. Resulting sequences were deposited in GISAID (www.gisaid.org, accessed on 25 December 2024).

### 2.3. Immunological Assays

Serological assays were conducted to quantify SARS-CoV-2-specific IgG antibodies using stored plasma samples. Bead-based immunoassays were performed in the Clinical Microbiology Department at the Miguel Servet Hospital for total nucleocapsid (N) antibody detection using a commercial platform (Elecsys Anti-SARS-CoV-2; Roche Diagnostics, Mannheim, German). Results were reported as numeric cutoff index (COI) values.

Inflammatory cytokines (IL-6, LDH, D-dimer, alkaline phosphatase, and Troponin I) and acute-phase proteins (CRP, glucose, and GGT) were profiled using Luminex assays (Luminex Corporation, Austin, TX, USA) in the Clinical Biochemistry Department at the Miguel Servet Hospital. The selection of these biomarkers in this study is grounded in their established role in reflecting key physiological and immunological disruptions associated with obesity and COVID-19 severity. Thus, IL-6, a key cytokine in both obesity and severe COVID-19, was measured due to its role in exacerbating inflammation and contributing to poor outcomes, such as ARDS and multi-organ failure [25]. D-dimer levels were assessed as markers of hypercoagulability, a complication often observed in COVID-19 patients with a high BMI, linking obesity-related vascular and metabolic disruptions to thrombotic risk [26]. LDH and CRP were included as indicators of tissue damage and systemic inflammation, which are both correlated with COVID-19 severity [27]. Troponin I, a marker of cardiac injury, was analyzed given the cardiovascular complications associated with both obesity and COVID-19 [28]. Biomarkers such as glucose, ferritin, and GGT were evaluated to assess metabolic dysregulation, which is common in obesity and worsened during SARS-CoV-2 infection [29]. Finally, SARS-CoV-2-specific neutralizing antibodies against the nucleocapsid protein were quantified to study immune responses in obese individuals, who may exhibit altered antibody profiles due to chronic inflammation and immune dysregulation [30]. Together, these biomarkers provide a comprehensive overview of the inflammatory, metabolic, and cardiovascular disruptions underlying increased COVID-19 severity in obese patients.

### 2.4. Statistical Analysis

Genomic sequences were analyzed to identify mutations linked to an elevated BMI. Mutations were categorized as synonymous, missense, or stop-gain and classified by their minor allele frequency (MAF). Logistic regression models were used to determine associations between specific mutations and clinical outcomes such as hospitalization and mortality, calculating odds ratios (OR) and confidence intervals (CI). To control for confounding factors, we included key variables such as age, gender and BMI in our logistic regression models. These covariates were selected based on their known influence on COVID-19 outcomes and obesity-related pathophysiology. No missing data were identified in the dataset, ensuring the robustness of the analyses. All statistical analyses were performed using R statistical software v.4.1.2, with *p*-values < 0.05 considered significant.

## 3. Results

### 3.1. Patient Characteristics and SARS-CoV-2 Clade Distribution in the Pre-Vaccine Era

**Figure 1:**
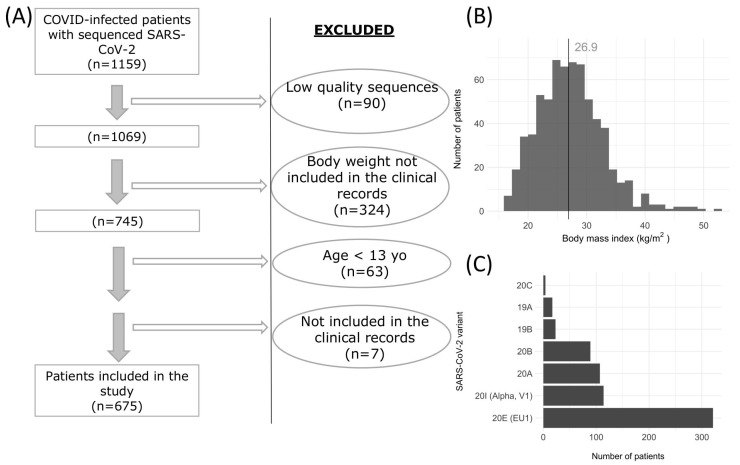
(**A**) Flow diagram of the COVID-19 patient screening and selection process, detailing the exclusion criteria applied for the final cohort selection. (**B**) Histogram showing the Body Mass Index (BMI) distribution among the included patients. (**C**) Bar plot representing the number of distinct SARS-CoV-2 variants analyzed, indicating the diversity of viral strains present in the study population.

Out of 1159 screened patients, 675 met the inclusion criteria (Figure 1A). Of these, 395 (58.5%) were women with an average age of 61 years, 260 (38.5%) required hospitalization, 24 (3.6%) were admitted to the ICU, and 95 (14.1%) died. The median BMI of the cohort was 26.9 kg/m^2^, as illustrated in Figure 1B.

Using Nextclade, we identified 205 mutations relative to the SARS-CoV-2 reference genome (Figure 1C), illustrating the virus’s evolution over time. The early clades 19A (2.52% of cases in this study) and 19B (3.41%) are foundational strains that were circulating globally in early 2020. Clades 20A (15.9%) and 20B (13.2%) emerged shortly after, carrying the D614G mutation, which increased transmissibility. Clade 20E (EU1) was the most prevalent in the cohort, comprising 47.6% of cases, and originated in Spain in mid-2020, spreading widely across Europe. Clade 20I (Alpha, V1) represented 16.9% of cases, marking the spread of the Alpha variant in late 2020 and early 2021. These percentages reflect the clade distribution within the study population, indicating temporal and regional shifts in viral evolution in SARS-CoV-2. Importantly, these variants circulated before vaccines were available, reflecting viral adaptations and spread patterns prior to immunization efforts.

### 3.2. Identification of BMI-Associated Viral Mutations

Out of the 205 identified mutations 205, 119 (58.3%) were missense, 77 (37.7%) were synonymous, and 3 (1.47%) were stop-gain mutations. Further analysis identified six mutations that were significantly associated with an elevated BMI. These included three synonymous mutations (C313T, C14599T, and A20268G), two missense mutations (C25904T and T22882G), and one stop-gain mutation (G28083T). Each mutation was located in the coding regions of specific viral proteins: C25904T in orf3A, C313T in nsp1 (part of orf1ab), T22882G in the spike protein, G28083T in orf8, C14599T in nsp12 (part of orf1ab), and A20268G in nsp15 (also part of orf1ab). Five mutations were classified as low-frequency variants (0.01 ≤ MAF <0.05), while A20268G was common (MAF > 0.05). Table 1 summarizes the BMI values of individuals carrying these mutations, along with the corresponding viral segments and proteins they encode.

### 3.3. Impact of Mutations on Clinical Outcomes

**Figure 2:**
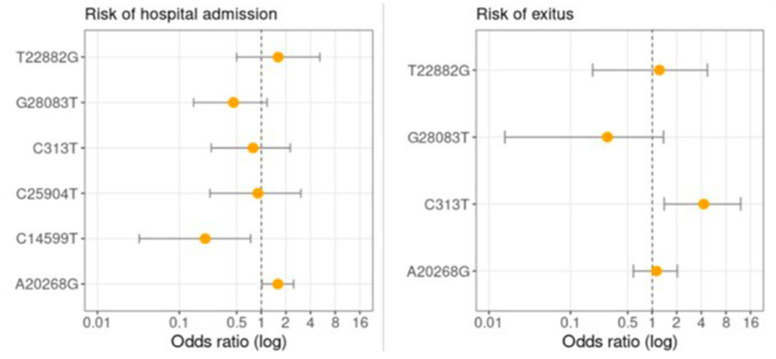
Logistic regression analysis showing the adjusted odds ratios (OR) and 95% confidence intervals for hospitalization and mortality associated with key SARS-CoV-2 mutations found in patients with an elevated BMI.

The logistic regression analysis was adjusted for age and gender and revealed that several viral mutations were associated with clinical outcomes, particularly hospitalization and mortality (Figure 2). The A20268G mutation, which was more common among patients with a higher BMI, increased the risk of hospitalization by approximately 59% (OR: 1.59; 95% CI: 1.02–2.49), indicating a potential risk factor for severe disease in this population. The C14599T mutation, also more frequently observed in individuals with an elevated BMI, appeared protective against hospitalization, with an odds ratio of 0.21 (95% CI: 0.03–0.74), suggesting a lower likelihood of requiring hospital care.

In terms of mortality, the C313T mutation, prevalent in patients with a higher BMI, showed a strong association with an increased risk of death, with a 4.28-fold higher mortality risk (95% CI: 1.40–12.15), marking it as a potential indicator of poor prognosis in this subgroup. These results highlight that mutations more prevalent in patients with an elevated BMI may influence clinical outcomes, with certain mutations potentially exacerbating the severity of COVID-19 in this high-risk group.

### 3.4. Immune Response Profiling: Association of SARS-CoV-2 Mutations with Neutralizing Antibody Levels Against the Nucleocapsid Protein

**Figure 3:**
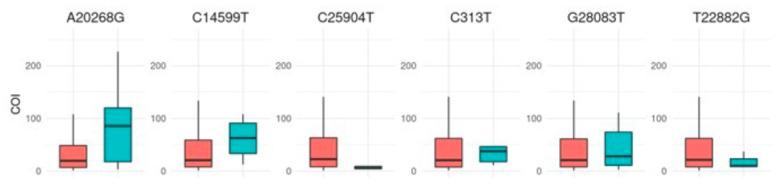
Boxplots showing the concentration of total neutralizing antibodies in plasma according to the presence (turquoise) or absence (red) of specific SARS-CoV-2 mutations. Antibody levels are reported as numeric values in the form of a cutoff index (COI; signal sample/cutoff).

An immunoassay measuring total SARS-CoV-2 antibodies (IgG, IgM, and IgA) against the nucleocapsid protein (Figure 3) showed no correlation between BMI and antibody levels. The assay employed a recombinant nucleocapsid (N) antigen in a double-antigen sandwich format, as described in the Methods section. However, patients carrying the A20268G mutation displayed significantly elevated levels of neutralizing antibodies (*p* < 0.001), suggesting a potentially enhanced immune response linked to this mutation. Other mutations showed no significant impact on antibody levels.

### 3.5. Inflammatory Markers and Clinical Parameters

**Figure 4:**
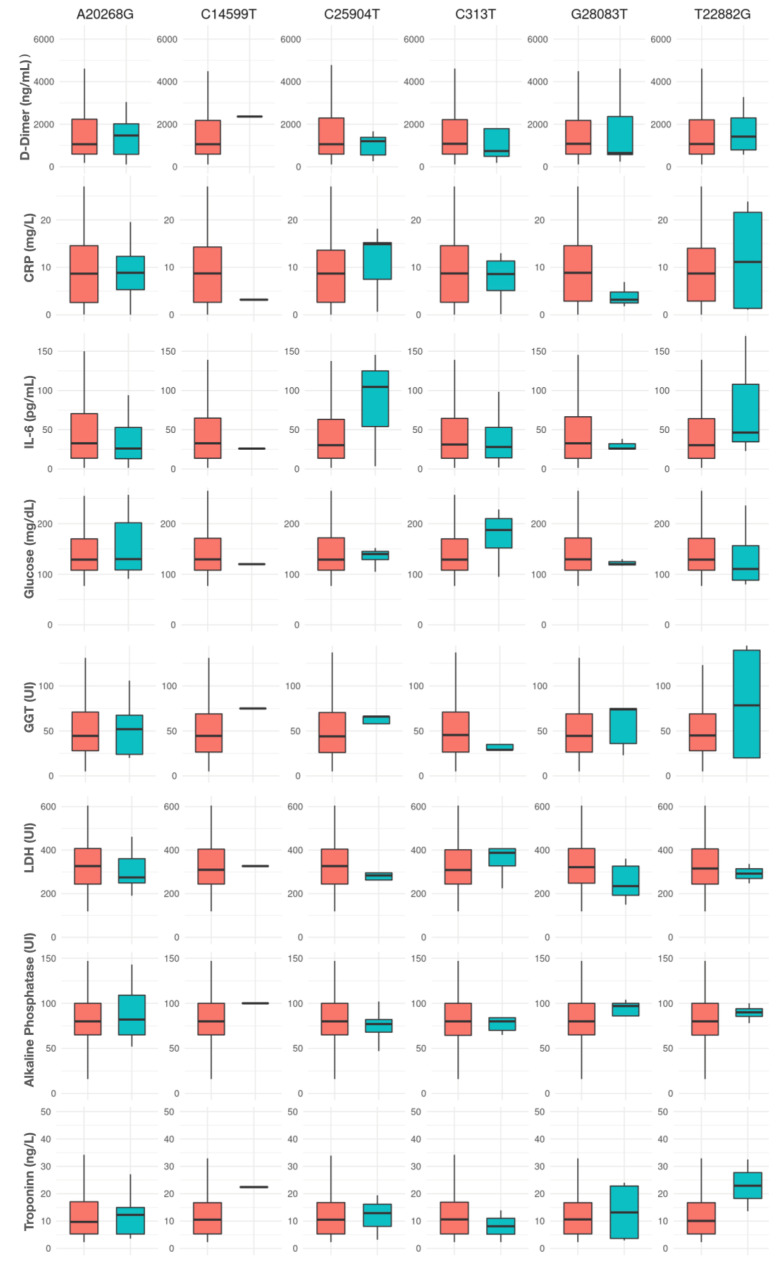
Inflammatory markers and cytokines in plasma according to the presence (turquoise) or absence (red) of specific SARS-CoV-2 mutations.

The analysis of plasma inflammatory markers based on SARS-CoV-2 mutations revealed certain trends in the inflammatory response profiles, though the overall results did not reach statistical significance (Figure 4).

For the A20268G mutation, carriers exhibited higher median levels of D-dimer (1886 ng/mL) compared with non-carriers (1241 ng/mL), along with slightly lower IL-6 levels (38.42 vs. 38.87 pg/mL). Levels of C-reactive protein (CRP), LDH, alkaline phosphatase, and Troponin I showed minimal variations based on the presence of this mutation.

The C14599T mutation was present in only one patient, whose inflammatory profile differed markedly from non-carriers, with lower CRP (3.19 vs. 9.11 mg/L), IL-6 (25.96 vs. 39.31 pg/mL), and LDH (327 vs. 339 UI) levels alongside slightly elevated D-dimer, ferritin, alkaline phosphatase, and Troponin I levels. These results should be interpreted with caution given the single occurrence of this mutation in the cohort.

In patients carrying viruses with the C25904T mutation, median IL-6 levels were notably higher (125.0 vs. 38.41 pg/mL in non-carriers), suggesting a possible trend (*p* = 0.27). Additionally, mutation carriers had elevated CRP levels (14.9 vs. 9.05 mg/L) and lower LDH levels (284.0 vs. 342.0 UI). D-dimer, alkaline phosphatase, and Troponin I levels showed no clear pattern.

For the C313T mutation, a slight decrease was observed in CRP (8.6 vs. 9.11 mg/L) and D-dimer (744.0 vs. 1314.5 ng/mL) levels in carriers, while IL-6, ferritin, LDH, alkaline phosphatase, and Troponin I levels remained relatively similar between groups.

The G28083T mutation displayed possible trends in both CRP and LDH levels, with carriers presenting lower median CRP (3.19 mg/L) and LDH (235 UI) levels compared with non-carriers (9.25 mg/L and 342 UI, respectively) (*p* = 0.07 and 0.06, respectively). Other markers, such as D-dimer, IL-6, alkaline phosphatase, and Troponin I, did not show notable differences.

For the T22882G mutation, carriers had higher median levels of CRP (11.15 vs. 9.11 mg/L), D-dimer (1420.5 vs. 1310.0 ng/mL), IL-6 (46.4 vs. 38.43 pg/mL), and Troponin I (22.9 vs. 12.55 ng/L) than non-carriers, although no strong trends were observed across these markers.

## 4. Discussion

In our study, we identified six SARS-CoV-2 mutations significantly associated with an elevated BMI that were linked to distinct clinical outcomes. The C313T mutation correlated with a marked increase in mortality risk, while A20268G was associated with a higher risk of hospitalization and elevated antibody levels, potentially indicating an adaptive immune response. Conversely, the C14599T mutation appeared to be protective against hospitalization. The trends in inflammatory markers associated with some mutations, particularly G28083T and C25904T, suggest an impact on the immune response. These findings highlight the complex interplay between viral genetics and host factors in COVID-19 severity among individuals with a high BMI.

The demographic profile of the cohort, with a median age of 61 years and an average BMI of 26.9 kg/m^2^, aligns with well-established risk factors for severe COVID-19 outcomes [31,32,33]. The high proportion of hospitalizations (38.5%) and mortality (14.1%) observed in our sample reinforces the vulnerability of individuals with elevated BMI, highlighting obesity as a significant risk factor for adverse outcomes.

With the identification of an increasing number of SARS-CoV-2 variants, research is shifting to the specific impacts of individual mutations and their combinations on disease progression and the immune response [24,34]. Notably, the C14599T mutation found in our study has also been documented in the XE recombinant variant, first identified in the UK in 2022 [35]. This variant, a recombination of the Omicron BA.1 and BA.2 subvariants, has raised concerns regarding transmissibility and immune escape [36]. Moreover, XE may exhibit different susceptibilities to the immunity conferred by current vaccines due to it having combined characteristics of BA.1 and BA.2.

Interestingly, in our cohort, C14599T appeared to be protective against hospitalization, which may initially seem to contrast with studies discussing its role in XE. This protective association might reflect unique factors within our cohort, such as BMI, comorbidities, or immune status, which may influence the role of C14599T in ways not directly comparable to its profile in XE. In addition, in XE, interactions with other mutations (e.g., BA.1 and BA.2 mutations) may amplify or alter its effects on transmissibility and the immune response. This underscores the possibility that C14599T’s impact depends on the broader mutational landscape. Furthermore, Jaros et al. [37] emphasize how the interplay between genetic predispositions and comorbidities, such as obesity, can significantly modify infection outcomes. Their study supports the therapeutic potential of addressing these comorbidities to mitigate the impact of SARS-CoV-2 infection, particularly in individuals with a predisposing genetic burden. Additionally, García-Lopez et al. [38] describe C14599T in combination with T7666C as characteristic of the BW.1 lineage, an Omicron subvariant originating in southeast Mexico. This highlights how local selection pressures can shape SARS-CoV-2 adaptability, underlining the significance of studying these mutations in different regional contexts.

Conversely, the A20268G mutation, a common variant (MAF > 0.05), was associated with an approximate 50% increase in hospitalization risk and significantly higher levels of neutralizing antibodies (*p* < 0.001). This fact points to a potentially adaptive immune response that may influence COVID-19 severity in patients with obesity. Research conducted by Liu et al. [39] identified this mutation as being prevalent in Spain (39%), Iceland (20%), and Scotland (20%), highlighting its spread in specific regions. Additionally, the A20268G mutation has been noted in prolonged SARS-CoV-2 infections within immunocompromised patients, as reported by Garcia-Vidal et al. [40], suggesting a role in viral persistence in certain immune-compromised environments. Individuals with obesity, characterized by chronic inflammation and immune dysregulation, may similarly provide a favorable environment for such mutations. Abedi et al. [41] suggests that miRNA dynamics may contribute to the virus’s adaptability within specific host environments. The regulatory influence of miRNAs could theoretically support or hinder the effects of such mutations by modulating viral protein synthesis or immune responses, especially in individuals with chronic inflammation or immune dysregulation, like those with obesity. The A20268G mutation was also identified as a regional marker during an outbreak in Suceava (Romania), where it became prevalent within the local population under quarantine conditions [42]. This observation suggests that A20268G may contribute to viral persistence in environments with limited external viral influx but high local transmission.

With regard to the C313T mutation, this was associated with a 428% increase in mortality risk, marking it as a potential indicator of poor prognosis in this high-risk group. This observation aligns with findings by Nagpal et al. [43], where C313T was identified by treeWAS as a mutation linked to severity. This mutation has also been noted frequently in India, often combined with C5700, suggesting potential regional adaptation [44]. The co-occurrence of C313T with C5700 may indicate a selective advantage in that specific geographic context, potentially driven by local host factors or population-specific pressures. Once again, this underscores how SARS-CoV-2 undergoes adaptation to local environments, influenced by region-specific factors and population dynamics. This highlights the importance of examining how regional or population-specific environments might shape the mutational landscape of SARS-CoV-2, as certain combinations of mutations could offer adaptive benefits that are not as evident when studied in isolation. Therefore, while C313T alone appears to correlate with higher mortality risk, its role in combination with other mutations, like C5700, could provide further insights into how SARS-CoV-2 adapts to different host populations.

Remarkably, all three mutations that were significantly associated with elevated BMI in our cohort were synonymous. Although synonymous mutations do not alter the amino acid sequence of viral proteins, they may still influence viral behavior in ways relevant to disease progression in individuals presenting obesity [45]. Synonymous mutations can impact the stability and translational efficiency of viral mRNA, potentially affecting the production rate and quantity of viral proteins. In the altered cellular environment typical of obesity, which is characterized by chronic inflammation and oxidative stress, even subtle changes in mRNA structure and translation may affect viral replication dynamics. It seems that SARS-CoV-2 may undergo selective pressures that favor certain synonymous mutations, allowing the virus to optimize its replication or persistence without modifying its protein structure. Indeed, research conducted by Ramazzotti et al. [46] demonstrated that synonymous mutations in SARS-CoV-2 can contribute significantly to the virus’s adaptation by optimizing codon usage to better match the host’s translational machinery. This codon optimization may improve the efficiency of viral protein synthesis, granting the virus a subtle yet meaningful advantage in human cells. In our cohort, the presence of synonymous mutations associated with an elevated BMI could similarly reflect a viral adaptation that enhances replication within the specific metabolic and inflammatory profile of individuals with obesity, which is supported by its occurrence on viral open reading frames and non-structural proteins. Recent studies indicate that while synonymous mutations might not drastically alter the viral RNA’s secondary structure, they can influence RNA stability and potentially impact replication rates [47].

Finally, regarding the other three mutations identified in our study (G28083T, C25904T, T22882G), these have not yet been directly associated with changes in disease severity in current studies. However, these mutations may contribute to the virus’s adaptation to new conditions and its spread within specific populations. In particular, the G28083T mutation could be linked to alterations in the spike protein, which is significant due to its crucial role in the virus’s infectivity, as observed in previous variants. On the other hand, the C25904T and T22882G mutations, while less studied, may be associated with modifications in other viral proteins that influence replication and the virus’s ability to evade the host’s immune response. Nevertheless, the absence of comprehensive clinical studies correlating these mutations with pathological effects in patients limits a definitive interpretation of their clinical relevance [48,49].

In summary, while the logistic regression analysis identified significant associations between specific viral mutations and clinical outcomes, particularly hospitalization and mortality, this study faced limitations, including a small sample size and some infinite confidence intervals. Therefore, validation in larger cohorts is essential to confirm these findings and to better understand the clinical implications of these mutations. Moreover, to mitigate selection bias, we screened all hospitalized COVID-19 patients during the study period and included only those with complete clinical and genetic data, ensuring a representative sample of the hospitalized population. We acknowledge that patients with incomplete records were excluded, which may introduce some bias; however, this decision was necessary to maintain data consistency for the statistical analyses. The analysis of plasma inflammatory markers and neutralizing antibody levels based on SARS-CoV-2 mutations revealed that certain mutations influenced immune and inflammatory responses. The A20268G mutation was associated with increased levels of neutralizing antibodies, suggesting an enhanced immune response in carriers. We hypothesize that this phenomenon is likely to be related to adaptations in the viral translational interaction with host proteins. These adaptations may influence antigen presentation by immune cells, thereby enhancing the immune response. While no direct correlation was found between BMI and antibody levels, this mutation’s association with higher antibody levels highlights a potential link with immune activation in specific patient subsets. For inflammatory markers, the G28083T mutation was associated with decreased levels of CRP and LDH, showing a trend (*p* < 0.1), whereas the C25904T mutation showed a trend toward increased IL-6 levels, which could suggest mutation-related impacts on inflammation, albeit without statistical significance. This is consistent with other studies that report elevated IL-6, D-dimer, LDH, and CRP levels in severe COVID-19 cases, independently of specific viral mutations [50]. The elevated inflammatory markers observed in cases with the T22882G mutation may be attributed to direct alterations in the immune response to the virus, as this mutation is a nonsynonymous change located in the spike protein. Moreover, this study provides evidence of the early emergence of this mutation in the virus’s evolutionary trajectory, suggesting that it became fixed in later variants associated with increased transmissibility. Such findings underscore that while these markers are useful indicators of severe inflammation, they may not necessarily correlate with particular genetic changes in the virus, thus highlighting again the complexity of the host–pathogen interaction and the predominantly host-driven nature of inflammatory responses in severe COVID-19.

This study emphasizes the significance of mutations in genes encoding non-structural proteins, as they could act as surrogate markers for diverse clinical outcomes linked to host–virus interactions. Such interactions may also involve the emergence of mutations in structural proteins with very low allelic frequencies or even viral quasispecies. These variants, often arising during prolonged infections, are difficult to detect using standard sequencing methods.

Given the increased risk of severe infection and mortality from COVID-19 in individuals with obesity, along with potential challenges in vaccine efficacy and breakthrough infections, further research is essential to develop tailored treatment plans and vaccine schedules that ensure durable, protective immune responses in this high-risk group. Future studies should aim to clarify the mechanisms linking obesity with SARS-CoV-2 mutation dynamics, examining how these mutations may influence disease progression, reinfection rates, and long-term immunity.

Given that this study was conducted on a specific hospitalized population in Spain, it is important to consider the generalizability of the findings to other settings. Factors such as variations in healthcare access, demographic characteristics, or the prevalence of obesity in different regions may affect the observed associations between SARS-CoV-2 mutations and clinical outcomes. These contextual differences should be taken into account when interpreting the applicability of our results to other populations or healthcare systems. Additionally, longitudinal studies across diverse populations with varying comorbidities would provide a more comprehensive understanding of how obesity and related conditions shape viral evolution and impact clinical outcomes.

## 5. Conclusions

This study identified mutations C14599, C313T, and A20268G as being significantly associated with a higher BMI. This fact suggests a potential link between these mutations and increased COVID-19 severity in patients with obesity and underscores the need for tailored clinical strategies.

Our findings also highlight the importance of studying SARS-CoV-2 mutations within a broader context, rather than in isolation, taking into account comorbidities, interactions with other mutations, and regional factors.

Further research in larger cohorts is essential to confirm these findings and to fully understand the implications of these mutations in the context of COVID-19 and obesity.

## Figures and Tables

**Table 1:** SARS-CoV-2 mutations associated with elevated BMI in the study cohort.

Mutation	Mutation Type	Viral Segment	Median BMI of Carriers	Number of Cases	Minor Allele Frequency (MAF)
C25904T	Missense	orf3A	32.00	11	0.02
C313T	Synonymous	nsp1 (orf1ab)	29.30	15	0.02
T22882G	Missense	spike	29.26	12	0.02
G28083T	Stop-gain	orf8	29.22	22	0.03
C14599T	Synonymous	nsp12 (orf1ab)	29.05	17	0.03
A20268G	Synonymous	nsp15 (orf1ab)	28.04	91	0.13

## Data Availability

The data presented in this study are available on request from the corresponding author upon Ethical Committee approval (CEICA, https://www.iacs.es/investigacion/comite-de-etica-de-la-investigacion-de-aragon-ceica/ceica-evaluaciones-y-otras-presentaciones/, accessed on 26 December 2024).
